# Supplementary material for: Lived experience of cognitive-communication changes for people with acquired brain injury and familiar communication partners: A qualitative evidence synthesis
Source: PLoS One. 2026 May 15;21(5):e0349220. doi: 10.1371/journal.pone.0349220 (PMC13178896; doi:10.1371/journal.pone.0349220)
Supplement: S3 File — (DOCX) [file pone.0349220.s003.docx]

Supplementary Material. Exclusion Reasons

| Authors | Year | Title | Exclusion reason |
| --- | --- | --- | --- |
| Matérne, M., Lundqvist, L. O., Strandberg, T. | 2017 | Opportunities and barriers for successful return to work after acquired brain injury: A patient perspective | Intervention study |
| Lexell, E. M. and Alkhed, A. K. and Olsson, K. | 2013 | The group rehabilitation helped me adjust to a new life: experiences shared by persons with an acquired brain injury | Intervention study |
| Gelech, J. and Bayly, M. and Desjardins, M. | 2019 | Constructing robust selves after brain injury: positive identity work among members of a female self-help group | Intervention study |
| Dixo, G. and Thornton, E. W. and Young, C. A. | 2007 | Perception of self-efficacy and rehabiltation among neurologically disabled adults | Intervention study |
| Mealings, M. and Douglas, J. and Olver, J. | 2015 | Beyond academic performance: Practice implications for working with students following traumatic brain injury | Not adult |
| Toma, C. L. and Hwang, J. and Kakonge, L. and Morrow, E. L. and Turkstra, L. S. and Mutlu, B. and Duff, M. C. | 2024 | Does Facebook Use Provide Social Benefits to Adults with Traumatic Brain Injury? | Not qualitative |
| O'Connell, K. and Marsh, A. A. and Edwards, D. F. and Dromerick, A. W. and Seydell-Greenwald, A. | 2022 | Emotion recognition impairments and social well-being following right-hemisphere stroke | Not qualitative |
| Gans, S. D. and Michaels, E. and Thaler, D. E. and Leung, L. Y. | 2022 | Detection of symptoms of late complications after stroke in young survivors with active surveillance versus usual care | Not qualitative |
| Girardi, G. and Farnese, M. L. and Scarponi, F. and De Tanti, A. and Bartolo, M. and Intiso, D. and Formisano, R. and Antonucci, G. | 2021 | User-centered practices in the eyes of informal caregivers of in-patients with severe acquired brain injury: needs, caring experience, and satisfaction | Not qualitative |
| Hewetson, R. and Cornwell, P. and Shum, D. | 2018 | Social participation following right hemisphere stroke: influence of a cognitive-communication disorder | Not qualitative |
| La Corte, V. and Serra, M. and George, N. and Pradat-Diehl, P. and Dalla Barba, G. | 2016 | Different patterns of recollection impairment in confabulation reveal different disorders of consciousness: A multiple case study | Not qualitative |
| Bellon, M. L. and Rees, R. J. | 2006 | The effect of context on communication: A study of the language and communication skills of adults with acquired brain injury | Not qualitative |
| Grayson, L. and Brady, M. C. and Togher, L. and Ali, M. | 2020 | A survey of cognitive–communication difficulties following TBI: are families receiving the training and support they need? | Not qualitative |
| Fadyl, J. K. and Payne, D. | 2016 | (Re)constructing identity following acquired brain injury: The complex journey of recovery after stroke | Not related to communication, experiences and/or impact |
| Lond, Benjamin J. and Williamson, Iain R. | 2022 | Acceptance, grief and adaptation amongst caregivers of partners with acquired brain injury: an interpretative phenomenological enquiry | Not related to communication, experiences and/or impact |
| Souesme, Guillaume, Poulin, Valérie, Ethier, Agnès, Grenier, Marianne, Sirois, Marie-Josée, Beaulieu-Bonneau, Simon, De Guise, Élaine, Lamontagne, Marie-Eve, Hudon, Carol, Émond, Marcel, Ouellet, Marie-Christine | 2024 | Challenges and facilitators in the experience of caregiving for an older adult with traumatic brain injury: A longitudinal qualitative study in the first-year postinjury | Not related to communication, experiences and/or impact |
| Lyon, Ionie and Fisher, Paul and Gracey, Fergus | 2021 | "Putting a new perspective on life": a qualitative grounded theory of posttraumatic growth following acquired brain injury | Not related to communication, experiences and/or impact |
| Dwyer, Aoife, Heary, Caroline, Ward, Marcia, MacNeela, Pádraig | 2019 | Adding insult to brain injury: young adults' experiences of residing in nursing homes following acquired brain injury | Not related to communication, experiences and/or impact |
| Martin, Rachelle and Levack, William M. M. and Sinnott, K. Anne | 2015 | Life goals and social identity in people with severe acquired brain injury: an interpretative phenomenological analysis | Not related to communication, experiences and/or impact |
| Kent, James and Wright-St Clair, Valerie A. and Kersten, Paula | 2014 | Older adults' experiences of community integration following traumatic brain injury | Not related to communication, experiences and/or impact |
| Fraas, M. R. and Calvert, M. | 2009 | The use of narratives to identify characteristics leading to a productive life following acquired brain injury | Not related to communication, experiences and/or impact |
| Hoogerdijk, Barbara and Runge, Ulla and Haugboelle, Jette | 2011 | The adaptation process after traumatic brain injury An individual and ongoing occupational struggle to gain a new identity | Not related to communication, experiences and/or impact |
| Lindén, A., Lexell, J., Lund, M. L. | 2010 | Perceived difficulties using everyday technology after acquired brain injury: Influence on activity and participation | Not related to communication, experiences and/or impact |
| Takada, K. and Sashika, H. and Wakabayashi, H. and Hirayasu, Y. | 2016 | Social participation and quality-of-life of patients with traumatic brain injury living in the community: A mixed methods study | Not related to communication, experiences and/or impact |
| Soeker, M. S. and Van Rensburg, V. and Travill, A. | 2012 | Individuals with traumatic brain injuries perceptions and experiences of returning to work in South Africa | Not related to communication, experiences and/or impact |
| Sacher, M. and Meixensberger, J. and Krupp, W. | 2018 | Interaction of quality of life, mood and depression of patients and their informal caregivers after surgical treatment of high-grade glioma: a prospective study | Not related to communication, experiences and/or impact |
| Rutz Voumard, R. and Kiker, W. A. and Dugger, K. M. and Engelberg, R. A. and Borasio, G. D. and Curtis, J. R. and Jox, R. J. and Creutzfeldt, C. J. | 2021 | Adapting to a New Normal After Severe Acute Brain Injury: An Observational Cohort Using a Sequential Explanatory Design | Not related to communication, experiences and/or impact |
| Pérez-de la Cruz, S. | 2022 | Perceptions of recovery and rehabilitation in people with brain injury in Spain. A qualitative study | Not related to communication, experiences and/or impact |
| Lennon, A., Bramham, J., Carroll, À, McElligott, J., Carton, S., Waldron, B., Fortune, D., Burke, T., Fitzhenry, M., Benson, C. | 2014 | A qualitative exploration of how individuals reconstruct their sense of self following acquired brain injury in comparison with spinal cord injury | Not related to communication, experiences and/or impact |
| King, P. R. and Beehler, G. P. and Vest, B. M. and Donnelly, K. and Wray, L. O. | 2018 | Qualitative exploration of traumatic brain injury-related beliefs among U.S. military veterans | Not related to communication, experiences and/or impact |
| Gosling, J. and Oddy, M. | 1999 | Rearranged marriages: marital relationships after head injury | Not related to communication, experiences and/or impact |
| Chamberlain, D. J. | 2006 | The experience of surviving traumatic brain injury | Not related to communication, experiences and/or impact |
| Kersey, J. and R. McArthur, A. and Hammel, J. | 2024 | Ongoing Challenges Related to the COVID-19 Pandemic Among People With Brain Injury | Not related to communication, experiences and/or impact |
| Nicklin, E. and Phang, I. and Short, S. C. and Hoogendoorn, P. and Boele, F. W. | 2023 | Patient and caregiver return to work after a primary brain tumor | Not related to communication, experiences and/or impact |
| Zanotto, A. and Goodall, K. and Ellison, M. and McVittie, C. | 2023 | Perceptions of social support and relationships while living with a brain tumour: a qualitative study | Not related to communication, experiences and/or impact |
| Ghosh-Cannell, C. and Fisher, P. and Ajayi, J. and Gracey, F. | 2023 | The experiences of wives following acquired brain injury (ABI). A qualitative analysis exploring realisations of change following the ABI of a "loved one" | Not related to communication, experiences and/or impact |
| Gould, K. R. and Carminati, J. Y. J. and Ponsford, J. L. | 2023 | "They just say how stupid I was for being conned". Cyberscams and acquired brain injury: A qualitative exploration of the lived experience of survivors and close others | Not related to communication, experiences and/or impact |
| Hänninen, V., Pohjola, H. | 2023 | Two Athletes’ Narratives of Traumatic Brain Injury, Identity, and Life Change | Not related to communication, experiences and/or impact |
| Karpa, J. V. | 2021 | Narrative Inquiry Methodology and Family Research: An Innovative Approach to Understanding Acquired Brain Injuries | Not related to communication, experiences and/or impact |
| Mealings, M. and Douglas, J. and Olver, J. | 2020 | Is it me or the injury: Students‚Äô perspectives on adjusting to life after traumatic brain injury through participation in study | Not related to communication, experiences and/or impact |
| Walker, J. and Schlebusch, L. and Gaede, B. | 2020 | The impact of stress on depression, ill health and coping in family members caring for patients with acquired brain injury | Not related to communication, experiences and/or impact |
| MacQueen, R. and Fisher, P. and Williams, D. | 2020 | A qualitative investigation of masculine identity after traumatic brain injury | Not related to communication, experiences and/or impact |
| Raanaas, R. K., Lund, A., Sveen, U., Asbjørnslett, M. | 2019 | Re-creating self-identity and meaning through occupations during expected and unexpected transitions in life | Not related to communication, experiences and/or impact |
| Holloway, M. and Orr, D. and Clark-Wilson, J. | 2019 | Experiences of challenges and support among family members of people with acquired brain injury: a qualitative study in the UK | Not related to communication, experiences and/or impact |
| Glintborg, C. and Thomsen, A. S. and Hansen, T. G. B. | 2018 | Beyond Broken Bodies and Brains: A Mixed Methods Study of Mental Health and Life Transitions after Brain Injury | Not related to communication, experiences and/or impact |
| Fadyl, J. K. and Payne, D. | 2016 | Socially constructed "value" and vocational experiences following neurological injury | Not related to communication, experiences and/or impact |
| Webster, J. and Taylor, A. and Balchin, R. | 2015 | Traumatic brain injury, the hidden pandemic: A focused response to family and patient experiences and needs | Not related to communication, experiences and/or impact |
| Alston, M. and Jones, J. and Curtin, M. | 2012 | Women and Traumatic Brain Injury: "It's not visible damage" | Not related to communication, experiences and/or impact |
| Lorenz, L. S. | 2010 | Discovering a new identity after brain injury | Not related to communication, experiences and/or impact |
| Brown, D. and Lyons, E. and Rose, D. | 2006 | Recovery from brain injury: Finding the missing bits of the puzzle | Not related to communication, experiences and/or impact |
| Jumisko, E., Lexell, J., Söderberg, S. | 2005 | The meaning of living with traumatic brain injury in people with moderate or severe traumatic brain injury | Not related to communication, experiences and/or impact |
| Nochi, M. | 2000 | Reconstructing self-narratives in coping with traumatic brain injury | Not related to communication, experiences and/or impact |
| Nochi, M. | 1997 | Dealing with the 'void': Traumatic brain injury as a story | Not related to communication, experiences and/or impact |
| Donker-Cools, B. H. P. M. and Schouten, M. J. E. and Wind, H. and Frings-Dresen, M. H. W. | 2018 | Return to work following acquired brain injury: the views of patients and employers | Not related to communication, experiences and/or impact |
| Carulli, L. A. and Degeneffe, C. E. and Olney, M. F. and Conrad, G. | 2018 | Social engagement among community college students with traumatic brain injury | Not related to communication, experiences and/or impact |
| Brassel, S. and Kenny, B. and Power, E. and Elbourn, E. and McDonald, S. and Tate, R. and MacWhinney, B. and Turkstra, L. and Holland, A. and Togher, L. | 2016 | Conversational topics discussed by individuals with severe traumatic brain injury and their communication partners during sub-acute recovery | Not related to communication, experiences and/or impact |
| Hall, A. and Grohn, B. and Nalder, E. and Worrall, L. and Fleming, J. | 2012 | A mixed methods study of the experience of transition to the community of working-aged people with non-traumatic brain injury | Not related to communication, experiences and/or impact |
| Yeates, G. and Henwood, K. and Gracey, F. and Evans, J. | 2007 | Awareness of disability after acquired brain injury and the family context | Not related to communication, experiences and/or impact |
| Bush, E. J. and Hux, K. and Guetterman, T. C. and McKelvey, M. | 2016 | The diverse vocational experiences of five individuals returning to work after severe brain injury: A qualitative inquiry | Not related to communication, experiences and/or impact |
| Jumisko, E., Lexell, J., Söderberg, S. | 2009 | The meaning of feeling well in people with moderate or severe traumatic brain injury | Not related to communication, experiences and/or impact |
| Armstrong, E. and Coffin, J. and Hersh, D. and Katzenellenbogen, J. M. and Thompson, S. C. and Ciccone, N. and Flicker, L. and Woods, D. and Hayward, C. and Dowell, C. and McAllister, M. | 2021 | “You felt like a prisoner in your own self, trapped”: the experiences of Aboriginal people with acquired communication disorders | Not related to communication, experiences and/or impact |
| Gould, K. R. and Hicks, A. J. and Hopwood, M. and Kenardy, J. and Krivonos, I. and Warren, N. and Ponsford, J. L. | 2019 | The lived experience of behaviours of concern: A qualitative study of men with traumatic brain injury | Not related to communication, experiences and/or impact |
| Saban, K. L. and Hogan, N. S. and Hogan, T. P. and Pape, T. L. B. | 2015 | He Looks Normal but… Challenges of Family Caregivers of Veterans Diagnosed with a Traumatic Brain Injury | Not related to communication, experiences and/or impact |
| Daggett, V. S. and Bakas, T. and Buelow, J. and Habermann, B. and Murray, L. L. | 2013 | Needs and concerns of male combat Veterans with mild traumatic brain injury | Not related to communication, experiences and/or impact |
| Atkin, K. and Stapley, S. and Easton, A. | 2010 | No one listens to me, nobody believes me: Self management and the experience of living with encephalitis | Not related to communication, experiences and/or impact |
| Gracey, F. and Palmer, S. and Rous, B. and Psaila, K. and Shaw, K. and O'Dell, J. and Cope, J. and Mohamed, S. | 2008 | "Feeling part of things": Personal construction of self after brain injury | Not related to communication, experiences and/or impact |
| Gifre, M. and Gil, A. and Pla, L. and Roig, T. and Monreal-Bosch, P. | 2015 | What happens after the accident? Psychosocial needs of people with traumatic brain injury and their families | Not related to communication, experiences and/or impact |
| Rimmer, B., Balla, M., Dutton, L., Lewis, J., Burns, R., Gallagher, P., Williams, S., Araújo-Soares, V., Finch, T., Sharp, L. | 2024 | "A Constant Black Cloud": The Emotional Impact of Informal Caregiving for Someone With a Lower-Grade Glioma | Not related to communication, experiences and/or impact |
| Keegan, L. C., Müller, N., Ball, M. J., Togher, L. | 2022 | Anger and aspirations: Linguistic analysis of identity after traumatic brain injury | Not related to communication, experiences and/or impact |
| Olofsson, A. and Larsson Lund, M. and Nyman, A. | 2020 | Everyday activities outside the home are a struggle: Narratives from two persons with acquired brain injury | Not related to communication, experiences and/or impact |
| Segev, E. and Levinger, M. and Hochman, Y. | 2018 | "Shared Destiny": The Dynamics of Relationships in Families of Patients With Brain Injury | Not related to communication, experiences and/or impact |
| Glintborg, C. | 2015 | Disabled & not normal: Identity construction after an acquired brain injury | Not related to communication, experiences and/or impact |
| Freeman, A. and Adams, M. and Ashworth, F. | 2015 | An exploration of the experience of self in the social world for men following traumatic brain injury | Not related to communication, experiences and/or impact |
| Hinckley, J. J. | 2014 | A case for the implementation of cognitive-communication screenings in acute stroke | Not related to communication, experiences and/or impact |
| Egbert, N. and Koch, L. and Coeling, H. and Ayers, D. | 2006 | The role of social support in the family and community integration of right-hemisphere stroke survivors | Not related to communication, experiences and/or impact |
| Petrella, L. and McColl, M. A. and Krupa, T. and Johnston, J. | 2005 | Returning to productive activities: Perspectives of individuals with long-standing acquired brain injuries | Not related to communication, experiences and/or impact |
| Adshead, C. D. and Norman, A. and Holloway, M. | 2021 | The inter-relationship between acquired brain injury, substance use and homelessness; the impact of adverse childhood experiences: an interpretative phenomenological analysis study | Not related to communication, experiences and/or impact |
| Godwin, E. and Chappell, B. and Kreutzer, J. | 2014 | Relationships after TBI: A grounded research study | Not related to communication, experiences and/or impact |
| Stein, M. S. and Reynolds, F. A. | 2022 | How is carer strain related to the recovery of stroke survivors with right hemisphere dysfunction? Implications for practice | Not related to communication, experiences and/or impact |
| Colantonio, Angela, Salehi, Sara, Kristman, Vicki, Cassidy, J. David, Carter, Angela, Vartanian, Oshin, Bayley, Mark, Kirsh, Bonnie, Hébert, Debbie, Lewkoh, John, Kubrak, Olena, Mantis, Steve, Vernich, Lee | 2016 | Return to work after work-related traumatic brain injury | Not specific inclusion of people with CCD (or its equivalent) |
| Conneeley, Anne Louise | 2002 | Social Integration following Traumatic Brain Injury and Rehabilitation | Not specific inclusion of people with CCD (or its equivalent) |
| Hammond, F. M. and Davis, C. S. and Hirsch, M. A. and Snow, J. M. and Kropf, M. E. and Schur, L. and Kruse, D. and Ball, A. M. | 2021 | Qualitative Examination of Voting Empowerment and Participation Among People Living With Traumatic Brain Injury | Not specific inclusion of people with CCD (or its equivalent) |
| Lefkovits, A. M. and Hicks, A. J. and Downing, M. and Ponsford, J. | 2021 | Surviving the "silent epidemic": A qualitative exploration of the long-term journey after traumatic brain injury | Not specific inclusion of people with CCD (or its equivalent) |
| Åke, S., Hartelius, L., Jakola, A. S., Antonsson, M. | 2023 | Experiences of language and communication after brain-tumour treatment: A long-term follow-up after glioma surgery | Not specific inclusion of people with CCD (or its equivalent) |
| Dinnes, C. and Hux, K. and Holmen, M. and Martens, A. and Smith, M. | 2018 | Writing changes and perceptions after traumatic brain injury: "oh, by the way, i can't write" | Not specific inclusion of people with CCD (or its equivalent) |
| Schipper, K. and Visser-Meily, J. M. and Hendrikx, A. and Abma, T. A. | 2011 | Participation of people with acquired brain injury: Insiders perspectives | Not specific inclusion of people with CCD (or its equivalent) |
| Gill, C. J. and Sander, A. M. and Robins, N. and Mazzei, D. K. and Struchen, M. A. | 2011 | Exploring experiences of intimacy from the viewpoint of individuals with traumatic brain injury and their partners | Not specific inclusion of people with CCD (or its equivalent) |
| Paniccia, A. and Colquhoun, H. and Kirsh, B. and Lindsay, S. | 2019 | Youth and young adults with acquired brain injury transition towards work-related roles: a qualitative study | Not specific inclusion of people with CCD (or its equivalent) |
| Knox, L. and Douglas, J. M. and Bigby, C. | 2015 | 'The biggest thing is trying to live for two people': Spousal experiences of supporting decision-making participation for partners with TBI | Not specific inclusion of people with CCD (or its equivalent) |
| Hammond, F. M. and Davis, C. S. and Cook, J. R. and Philbrick, P. and Hirsch, M. A. | 2012 | Relational dimension of irritability following traumatic brain injury: a qualitative analysis | Not specific inclusion of people with CCD (or its equivalent) |
| Carlozzi, N. E. and Kratz, A. L. and Sander, A. M. and Chiaravalloti, N. D. and Brickell, T. A. and Lange, R. T. and Hahn, E. A. and Austin, A. and Miner, J. A. and Tulsky, D. S. | 2015 | Health-related quality of life in caregivers of individuals with traumatic brain injury: development of a conceptual model | Not specific inclusion of people with CCD (or its equivalent) |
| Brown, A. and Barth, D. C. and Leslie, A. R. | 2024 | "You're Someone Different Now": An Autoethnography on Identity and Occupational Identity Disruption After Traumatic Brain Injury | Not specific inclusion of people with CCD (or its equivalent) |
| Lim, H. and Kakonge, L. and Hu, Y. and Turkstra, L. and Duff, M. and Toma, C. and Mutlu, B. | 2023 | So, I Can Feel Normal: Participatory Design for Accessible Social Media Sites for Individuals with Traumatic Brain Injury | Not specific inclusion of people with CCD (or its equivalent) |
| Dunne, S. and Williams, G. P. and Bradbury, C. and Keyes, T. and Lane, A. R. and Yang, K. and Ellison, A. | 2023 | Uncovering the social determinants of brain injury rehabilitation | Not specific inclusion of people with CCD (or its equivalent) |
| Hendryckx, C. and Couture, M. and Gosselin, N. and Nalder, E. and Gagnon-Roy, M. and Thibault, G. and Bottari, C. | 2024 | The dual reality of challenging behaviours: Overlapping and distinct perspectives of individuals with TBI and their caregivers | Not specific inclusion of people with CCD (or its equivalent) |
| Ahmadi, R. and Lim, H. and Mutlu, B. and Duff, M. and Toma, C. and Turkstra, L. | 2022 | Facebook Experiences of Users With Traumatic Brain Injury: A Think-Aloud Study | Not specific inclusion of people with CCD (or its equivalent) |
| Stagg, K. and Douglas, J. and Iacono, T. | 2023 | Living with stroke during the first year after onset: an instrumental case study exploring the processes that influence adjustment | Not specific inclusion of people with CCD (or its equivalent) |
| Davidson, C. S. and Wallace, S. E. | 2022 | Information needs for carers following a family member‚Äôs right hemisphere stroke | Not specific inclusion of people with CCD (or its equivalent) |
| Abraham, T. H. and Ono, S. S. and Moriarty, H. and Winter, L. and Bender, R. E. and Facundo, R. and True, G. | 2021 | Revealing the Invisible Emotion Work of Caregivers: A Photovoice Exploration of Informal Care Provided by Family Caregivers for Post-9/11 Veterans with Traumatic Brain Injuries | Not specific inclusion of people with CCD (or its equivalent) |
| Douglas, J. | 2020 | Loss of friendship following traumatic brain injury: A model grounded in the experience of adults with severe injury | Not specific inclusion of people with CCD (or its equivalent) |
| Analytis, P. and Warren, N. and Ponsford, J. | 2020 | The sibling relationship after acquired brain injury (ABI): perspectives of siblings with ABI and uninjured siblings | Not specific inclusion of people with CCD (or its equivalent) |
| O’Keeffe, F., Dunne, J., Nolan, M., Cogley, C., Davenport, J. | 2020 | "The things that people can't see" The impact of TBI on relationships: an interpretative phenomenological analysis | Not specific inclusion of people with CCD (or its equivalent) |
| Jumisko, E., Lexell, J., Söderberg, S. | 2007 | Living with moderate or severe traumatic brain injury: The meaning of family members' experiences | Not specific inclusion of people with CCD (or its equivalent) |
| Howes, H. and Benton, D. and Edwards, S. | 2005 | Women's experience of brain injury: An interpretative phenomenological analysis | Not specific inclusion of people with CCD (or its equivalent) |
| Cavers, D. and Hacking, B. and Erridge, S. E. and Kendall, M. and Morris, P. G. and Murray, S. A. | 2012 | Social, psychological and existential well-being in patients with glioma and their caregivers: a qualitative study | Not specific inclusion of people with CCD (or its equivalent) |
| Cooper, J. and Kierans, C. and Defres, S. and Easton, A. and Kneen, R. and Solomon, T. | 2017 | Care beyond the hospital ward: Understanding the socio-medical trajectory of herpes simplex virus encephalitis | Not specific inclusion of people with CCD (or its equivalent) |
| Egan, J. and Chenoweth, L. and McAuliffe, D. | 2006 | Email-facilitated qualitative interviews with traumatic brain injury survivors: A new and accessible method | Not specific inclusion of people with CCD (or its equivalent) |
| Edvardsson, T., Ahlström, G. | 2005 | Illness-related problems and coping among persons with low-grade glioma | Not specific inclusion of people with CCD (or its equivalent) |
| Walker, H., Rimmer, B., Dutton, L., Finch, T., Gallagher, P., Lewis, J., Burns, R., Araújo-Soares, V., Williams, S., Sharp, L. | 2023 | Experiences of work for people living with a grade 2/3 oligodendroglioma: a qualitative analysis within the Ways Ahead study | Not specific inclusion of people with CCD (or its equivalent) |
| Nichols, J. L. and Kosciulek, J. | 2014 | Social interactions of individuals with traumatic brain injury | Not specific inclusion of people with CCD (or its equivalent) |
| Douglas, J. M. | 2013 | Conceptualizing self and maintaining social connection following severe traumatic brain injury | Not specific inclusion of people with CCD (or its equivalent) |
| Salas, C. E. and Casassus, M. and Rowlands, L. and Pimm, S. and Flanagan, D. A. J. | 2018 | "Relating through sameness": a qualitative study of friendship and social isolation in chronic traumatic brain injury | Not specific inclusion of people with CCD (or its equivalent) |
| Knox, L. and Douglas, J. M. and Bigby, C. | 2017 | "I've never been a yes person": Decision-making participation and self-conceptualization after severe traumatic brain injury | Not specific inclusion of people with CCD (or its equivalent) |
| Landau, J. and Hissett, J. | 2008 | Mild Traumatic Brain Injury: Impact on Identity and Ambiguous Loss in the Family | Not specific inclusion of people with CCD (or its equivalent) |
| Wyse, J. J. and Pogoda, T. K. and Mastarone, G. L. and Gilbert, T. and Carlson, K. F. | 2020 | Employment and vocational rehabilitation experiences among veterans with polytrauma/traumatic brain injury history | Not specific inclusion of people with CCD (or its equivalent) |
| Morris, P. G. and Prior, L. and Deb, S. and Lewis, G. and Mayle, W. and Burrow, C. E. and Bryant, E. | 2005 | Patients' views on outcome following head injury: a qualitative study | Not specific inclusion of people with CCD (or its equivalent) |
| Fama ME, Schwartzman S, Metzler E, Coyle S, Hatfield B | 2025 | Self-reported longitudinal impacts of the COVID-19 pandemic on adults with acquired communication disorders. | Not specific inclusion of people with CCD (or its equivalent) |
| Donovan-Kicken, E. and Bute, J. J. | 2008 | Uncertainty of social network members in the case of communication-debilitating illness or injury | Qualitative data not reported separately |
| Wilson, B. J. and Bright, F. A. S. and Cummins, C. and Elder, H. and Kayes, N. M. | 2022 | 'The wairua first brings you together': Māori experiences of meaningful connection in neurorehabilitation | Wrong population |
| Piil, K. and Jakobsen, J. and Christensen, K. B. and Juhler, M. and Guetterman, T. C. and Fetters, M. D. and Jarden, M. | 2018 | Needs and preferences among patients with high-grade glioma and their caregivers - A longitudinal mixed methods study | Wrong population |
| Kattari, S. K. | 2014 | Sexual experiences of adults with physical disabilities: Negotiating with sexual partners | Wrong population |
| Salander, P. and Bergenheim, A. T. and Henriksson, R. | 2000 | How was life after treatment of a malignant brain tumour? | Wrong population |
| Kelly, C. and Cornwell, P. and Copley, A. and Hewetson, R. | 2022 | Community-based rehabilitation for adults with cognitive-communication disorders following traumatic brain injury: A mixed methods investigation | Wrong population |
| Elbourn, E. and Brassel, S. and Steel, J. and Togher, L. | 2023 | The lived experience of communication recovery from 6 months to 2 years after severe TBI | Wrong publication type |
| Reeder, S. and Moore, N. and Lannin, N. A. and Semple, B. D. | 2023 | 'Would you take a drug for this?' A qualitative study on social functioning in people with TBI and their attitude towards novel targeted treatments | Wrong publication type |
| Ahmadi, R. and Lim, H. and Mutlu, B. and Duff, M. and Turkstra, L. | 2023 | Facebook use by adults with TBI: A think-aloud study | Wrong publication type |
| Topping, M. and Douglas, J. and Winkler, D. | 2023 | "They treat you like a person, they ask you what you want": Quality support grounded in the lived experience of people with neurological disability, close others, and disability support workers | Wrong publication type |
| Mintah, K. and Desrocher, M. | 2021 | Qualitative Analyses of Cognitive and Social Difficulties Reported by Emerging Adults with Mild Traumatic Brain Injury | Wrong publication type |
| Brunner, M. and Palmer, S. and Togher, L. and Dann, S. and Hemsley, B. | 2023 | What's it like to use Twitter after a traumatic brain injury? | Wrong publication type |
| Armstrong, E. and Coffin, J. and Hersh, D. and Katzenellenbogen, J. M. and Thompson, S. C. and Ciccone, N. and Flicker, L. and Woods, D. and Hayward, C. and Meaghan, M. | 2023 | Aboriginal Australian experiences of brain injury and ways forward in culturally secure rehabilitation | Wrong publication type |
| Hewetson, R. and Cornwell, P. and Shum, D. | 2023 | Exploring social network maintenance and quality of life in people with social cognition impairment post-right hemisphere stroke | Wrong publication type |
