# Supplementary material for: Lived experience of cognitive-communication changes for people with acquired brain injury and familiar communication partners: A qualitative evidence synthesis
Source: PLoS One. 2026 May 15;21(5):e0349220. doi: 10.1371/journal.pone.0349220 (PMC13178896; doi:10.1371/journal.pone.0349220)
Supplement: S4 File — (DOCX) [file pone.0349220.s004.docx]

S4. GRADE-CERQual ratings for themes and sub-themes

GRADE- CERQual for sub-themes related to person with brain injury

| Study information | CERQual Quality Assessment | | | | |  |
| --- | --- | --- | --- | --- | --- | --- |
|  | Methodological limitations | Coherence of findings | Relevance of evidence | Adequacy of data | Overall confidence | |
| Sub-theme: Communicating is not easy | | | | | |  |
| 11 studies:   - Brunner et al., 2019 - Brunner et al., 2020 - Elbourn et al., 2022 - Grayson et al., 2021 - Kelly et al., 2022 - Norman et al., 2023 - O’Flaherty et al., 1997 - Shorland & Douglas, 2010 - Skromanis et al., 2025 - van den Broek et al., 2025 - VanSolkema et al., 2024 | No or very minor concerns | No or very minor concerns | No or very minor concerns | No or very minor concerns | HIGH | |
| Sub-theme: Lack of awareness and feeling tired | | | | | | |
| 11 studies:   - Bertram et al., 2019 - Brunner et al., 2019 - Brunner et al., 2020 - Elbourn et al., 2022 - Grayson et al., 2021 - Kelly et al., 2022 - Norman et al., 2023 - O’Flaherty et al., 1997 - Shorland & Douglas, 2010 - Skromanis et al., 2025 - VanSolkema et al., 2024 | No or very minor concerns | No or very minor concerns | No or very minor concerns | No or very minor concerns | HIGH | |
| Sub-theme: Anxiety, embarrassment and isolation | | | | | | |
| 12 studies:   - Armstrong et al., 2019 - Brunner et al., 2019 - Brunner et al., 2020 - Elbourn et al., 2022 - Grayson et al., 2021 - Kelly et al., 2022 - Norman et al., 2023 - O’Flaherty et al., 1997 - Shorland & Douglas, 2010 - Skromanis et al., 2025 - Van den Broek et al., 2025 - VanSolkema et al., 2024 | No or very minor concerns | No or very minor concerns | No or very minor concerns | No or very minor concerns | HIGH | |
| Sub-theme: Connecting with others | | | | | | |
| 11 studies:   - Armstrong et al., 2019 - Bertram et al., 2019 - Brunner et al., 2019 - Brunner et al., 2020 - Grayson et al., 2021 - Kelly et al., 2022 - Norman et al., 2023 - O’Flaherty et al., 1997 - Shorland & Douglas, 2010 - Skromanis et al., 2025 - VanSolkema et al., 2024 | No or very minor concerns | Minor concerns  (Evidence downgraded as three studies contained data with unexplained contradictions) | No or very minor concerns | No or very minor concerns | HIGH | |
| Sub-theme: Participation and identity | | | | | | |
| 11 studies:   - Armstrong et al., 2019 - Bertram et al., 2019 - Brunner et al., 2019 - Elbourn et al., 2022 - Grayson et al., 2021 - Kelly et al., 2022 - Norman et al., 2023 - O’Flaherty et al., 1997 - Shorland & Douglas, 2010 - Skromanis et al., 2025 - VanSolkema et al., 2024 | No or very minor concerns | No or very minor concerns | No or very minor concerns | No or very minor concerns | HIGH | |

GRADE- CERQual for sub-themes related to communication partner

| Study information | CERQual Quality Assessment | | | | |  |
| --- | --- | --- | --- | --- | --- | --- |
|  | Methodological limitations | Coherence of findings | Relevance of evidence | Adequacy of data | Overall confidence | |
| Sub-theme: Adjusting to increased support | | | | | |  |
| 7 studies:   - Bertram et al., 2019 - Grayson et al., 2021 - Kelly et al., 2022 - O’Flaherty et al., 1997 - Skromanis et al., 2025 - van den Broek et al., 2025 - VanSolkema et al., 2024 | Minor concerns  (Evidence downgraded due to minor concerns about methodological limitations in one study as per CASP qualitative checklist) | No or very minor concerns | No or very minor concerns | No or very minor concerns | HIGH | |
| Sub-theme: Emotional toll of support | | | | | | |
| 5 studies:   - Grayson et al., 2021 - Kelly et al., 2022 - O’Flaherty et al., 1997 - van den Broek et al., 2025 - VanSolkema et al., 2024 | Minor concerns  (Evidence downgraded due to minor concerns about methodological limitations in one study as per CASP qualitative checklist) | No or very minor concerns | No or very minor concerns | No or very minor concerns | HIGH | |
| Sub-theme: Relationship and life role changes | | | | | | |
| 5 studies:   - Grayson et al., 2021 - Kelly et al., 2022 - O’Flaherty et al., 1997 - van den Broek et al., 2025 - VanSolkema et al., 2024 | Minor concerns  (Evidence downgraded due to minor concerns about methodological limitations in one study as per CASP qualitative checklist) | No or very minor concerns | Minor concerns  (Evidence downgraded as findings derived from studies without rich data) | No or very minor concerns | MODERATE | |
