# Supplementary material for: Lived experience of cognitive-communication changes for people with acquired brain injury and familiar communication partners: A qualitative evidence synthesis
Source: PLoS One. 2026 May 15;21(5):e0349220. doi: 10.1371/journal.pone.0349220 (PMC13178896; doi:10.1371/journal.pone.0349220)
Supplement: Attachment — Submitted filename: Response to reviewers.docx [file pone.0349220.s005.docx]

**Response to reviews**

Thank you to the reviewers for their kind words and for taking the time to read this manuscript. We welcome your time, effort and thoughts.

| Editorial comments | Response to reviewers |
| --- | --- |
| Please ensure that your manuscript meets PLOS ONE's style requirements, including those for file naming | Done |
| Kindly update the title of your manuscript to include the phrase 'systematic review' in it. | We have left this as “qualitative evidence synthesis” as agreed by the journal. |
| Financial disclosure. Please state what role the funders took in the study. | Done |
| Please confirm at this time whether or not your submission contains all raw data required to replicate the results of your study. | All data is included as consistent with a qualitative systematic review. |
| Reviewer 1 |  |
| The authors may wish to consider include clarifying the ethics statement to explicitly note that ethical approval was not required due to secondary analysis of published data | Statement added in methods |
| Briefly acknowledging the predominance of Australian studies and heterogeneity in how cognitive-communication disorder was identified as potential limitations affecting transferability | Added in limitations |
| Reviewer 2 |  |
| I suggest revising the title to better reflect the stated objective, as the study focuses not only on individuals with ABI but also on their family members. | Title changed. |
| Adding more background on sociocultural and ecological factors would strengthen the context, as these factors may significantly influence cognitive-communication changes in this population. | Added |
| In the Methods section, please provide additional detail regarding how studies in different languages were handled during analysis, if applicable. | No studies from different languages were identified. |
| Clarification on the timeline and scope of the literature search would also be helpful. | The initial search was conducted in May 2024 and rerun in August 2025. This information is included in the methods, and “search strategy” section. |
| It is somewhat unclear why the inclusion criteria limited the research designs primarily to qualitative and mixed-methods studies. Further justification for this decision would improve clarity. | This review was focused on the lived experience of cognitive-communication changes, and *how* these changes influence others, rather than a quantitative systematic review. |
| Reviewer 3 |  |
| No comments to respond to |  |
